# Unintentional Jimson Weed Poisoning in a Family: A Case Report

**DOI:** 10.7759/cureus.45604

**Published:** 2023-09-20

**Authors:** Moutushi Ahmed, Debasish Mridha

**Affiliations:** 1 Internal Medicine, Khulna Medical College, Khulna, BGD; 2 Neurology, Michigan Advanced Neurology Center, Saginaw, USA

**Keywords:** tropane alkaloids, hallucinations, pregnant woman, homegrown produce, family ingestion, unintended exposure, anticholinergic effects, datura stramonium, jimson weed poisoning

## Abstract

This case report reveals a rare incident of unintended Jimson weed (*Datura stramonium*) exposure within a family in the United States. In this narrative, a pregnant 36-year-old Asian woman and her family unknowingly ingested homemade soup infused with Jimson weed leaves. This led to symptoms such as vomiting, dry mouth, blurred vision, flushed skin, breathing difficulties, and hallucinations. While the woman and her daughter quickly recovered, the husband's severe hallucinations required intensive care. The episode underscores the vital importance of accurate plant identification, particularly in homegrown produce. Recognizing and understanding anticholinergic poisoning symptoms becomes crucial for timely diagnosis and intervention, preventing such occurrences. This case serves as a poignant reminder of the potential risks concealed within our everyday environments.

## Introduction

Jimson weed (*Datura stramonium*), belonging to the *Solanaceae *family, is renowned for its abundance of tropane alkaloids, encompassing scopolamine, hyoscyamine, and atropine [1]. These alkaloids engage in competitive antagonism with acetylcholine at muscarinic receptors, eliciting a spectrum of anticholinergic effects [2]. While Jimson weed intoxication is prevalent in certain geographical pockets, documented cases within the United States are notably scarce [3]. This wild herb flourishes across the country, maturing between May and September. Occasionally, adolescents display curiosity regarding its hallucinogenic potential [4]. The emergence of home gardening introduces fresh complexities to the landscape of plant-related intoxications. Within this context, this case report offers insight into a unique episode involving inadvertent Jimson weed ingestion within a familial setting.

## Case presentation

The patient, along with her husband (42) and daughter (7), was transported urgently to the emergency department (ED) due to profound episodes of emesis, intense nausea, visual disturbances, skin flushing, and abnormal behavior characterized by distressing hallucinations. The swift response was initiated by a concerned neighbor who promptly contacted the mobile medical response (MMR) team. The patient was at 18 weeks of gestation and adhered to a health-conscious dietary regimen.

As per the medical history provided, on the evening in question, the woman prepared a home-cooked soup using vegetables and leaves harvested from her own backyard garden, including kale that she had planted herself. The entire family, including her husband, daughter, and herself, shared this meal together. It is worth noting that her daughter only had a very small portion of the soup. Roughly 30-40 minutes after consuming the meal, various symptoms began to manifest. Both the woman and her daughter experienced severe nausea, vomiting, and even hallucinations. The husband, while not vomiting as much, started to exhibit tactile and visual hallucinations. In the ED, the woman exhibited restlessness, accompanied by multiple bouts of vomiting and trembling. Despite being conscious and aware of her identity, she showed confusion regarding her location and the current time. During the examination, the patient engaged in peculiar actions, seemingly reaching out to grasp an object that was not present. Furthermore, she seemed to be conversing with an unseen entity, indicative of hallucinations.

Vital signs and parameters recorded at the ED are presented in Table 1.

**Table 1:** Vitals and parameters for patient and family at the emergency department.

Case	Blood Pressure	Heart Rate	Oral Temperature	Respiratory Rate	Pupil Size	Reaction to Light	Bowel Sounds	Reflexes	Skin Condition
Woman	125/85 mmHg	120 bpm	99.8 F (37.4 C)	20/min	7 mm	Reactive	Decreased	Normal	Warm
Husband	147/95 mmHg	132 bpm	101.2 F (38.4 C)	24/min	8 mm	Reactive	Decreased	Decreased	Warm
Daughter	115/75 mmHg	110 bpm	98.5 F (36.94 C)	18/min	6 mm	Reactive	Normal	Normal	Normal

After the initial assessment, preliminary treatment was started. Other diagnostic evaluations, encompassing fingerstick blood glucose, comprehensive blood count analysis, chemistry panel investigation, and urinalysis, all yielded results well within the expected ranges. Additionally, a toxicology screening revealed negative outcomes for common substances such as alcohol, benzodiazepines, amphetamines, marijuana, tricyclic antidepressants, opiates, and phencyclidine. Sinus tachycardia was observed in the electrocardiogram (EKG), without any identifiable anomalies. Although the woman and her daughter's condition was not alarming, the husband's arterial blood gas (ABG) analysis revealed abnormal findings. Furthermore, elevated CPK levels in renal findings raised concern for rhabdomyolysis, considering the husband's worsening condition, he was transferred to the intensive care unit (ICU) for closer monitoring and care.

Building upon the clinical presentation of the family and the history of the preparation of soup from backyard plants, the attending physician within the ED harbored suspicions of potential Jimson weed intoxication unknowingly. These suspicions were ultimately validated using a plant identification app, allowing for a direct comparison between the plant used in the soup preparation and images on the patient's phone. This innovative approach played a pivotal role in precisely diagnosing Jimson's weed intoxication.

After the confirmed diagnosis of Jimson weed poisoning, appropriate treatment interventions were swiftly executed. Given the family's delayed arrival (>one hour) at the ER following ingestion, the administration of activated charcoal was deemed unsuitable. Consequently, intravenous (IV) normal saline infusion was promptly initiated to maintain fluid balance.

In response to the female patient's pronounced agitation, an initial dose of 5 mg of lorazepam was administered intravenously to alleviate her heightened arousal. Considering her pregnancy, no antipsychotic medication was prescribed. Subsequently, two more doses of lorazepam were administered over a controlled timeframe to effectively manage her agitation. Following the stabilization of the patient's condition, a gestational ultrasound was conducted, yielding reassuring results that affirmed the health and viability of the fetus without any indications of toxicity. She was closely observed with continuous heart monitoring for the next 48 hours.

While the daughter remained stable, consistently exhibiting acceptable laboratory parameters, a cautious approach was undertaken, involving a 0.5 mg dose of lorazepam and diligent observation. In contrast, due to the husband's deteriorating state, a carefully administered combination of physostigmine (2 mg) and lorazepam (10 mg) was introduced via the IV route to counteract escalating symptoms. The physostigmine dose was repeated after 30 min. Gradually, his condition improved. Simultaneously, continuous intravenous solutions were administered, leading to a gradual decrease in CPK levels. On the second day, he was also prescribed clindamycin for aspiration pneumonia. His condition stabilized, and he was transferred to the medicine ward on the fourth day, where he remained for another two days under close observation. Given that he had consumed most of the soup, his recovery was slower. Eventually, he was discharged on the seventh day.

Following a concise four-day hospitalization, both the wife and daughter were discharged from medical care. The woman received appropriate guidance for her pregnancy and was discharged with an OB/GYN follow-up scheduled for the same week. Additionally, specialized consultations with neurology and psychology experts were proactively conducted to address any potential residual neurological or psychological ramifications.

## Discussion

**Figure 1:**
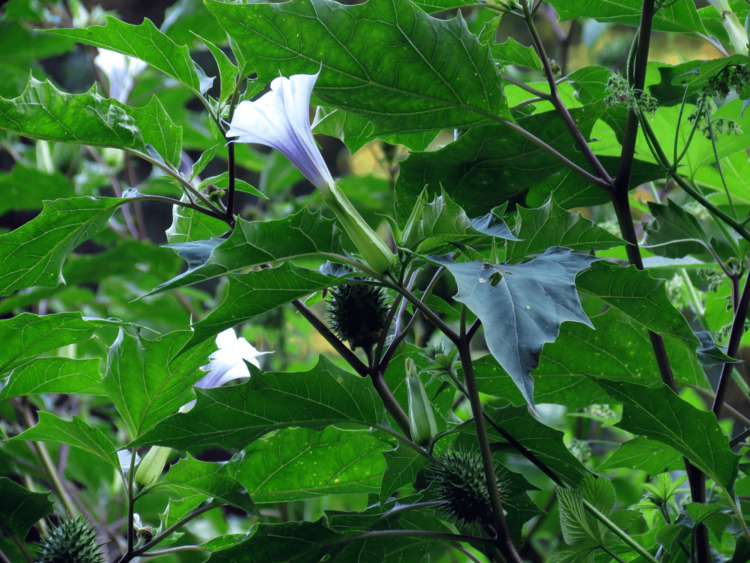
Jimson weed (Datura stramonium). This image is adapted from "Weeds of the South" by Charles T. Bryson and Michael S. DeFelice (2009), by University of Georgia Press (https://ugapress.org/book/9780820330464/weeds-of-the-south/).

Jimson weed, a member of the nightshade family (*Solanaceae*), gained historical significance as "Jamestown weed" due to its link to intoxication incidents in Jamestown, Virginia, in 1676 (Figure 1) [4]. Subsequently, its name was shortened to "jimsonweed." This plant is also recognized by various names, including thorn apple, angel's trumpet, stinkweed, and green dragon. Its use in traditional medicine over centuries for conditions, such as asthma, diarrhea, intestinal cramps, and nocturia, highlights its anticholinergic properties. Interestingly, even Homer's epic poem, *The Odyssey*, hints at its hallucinogenic effects [5].

**Figure 2:**
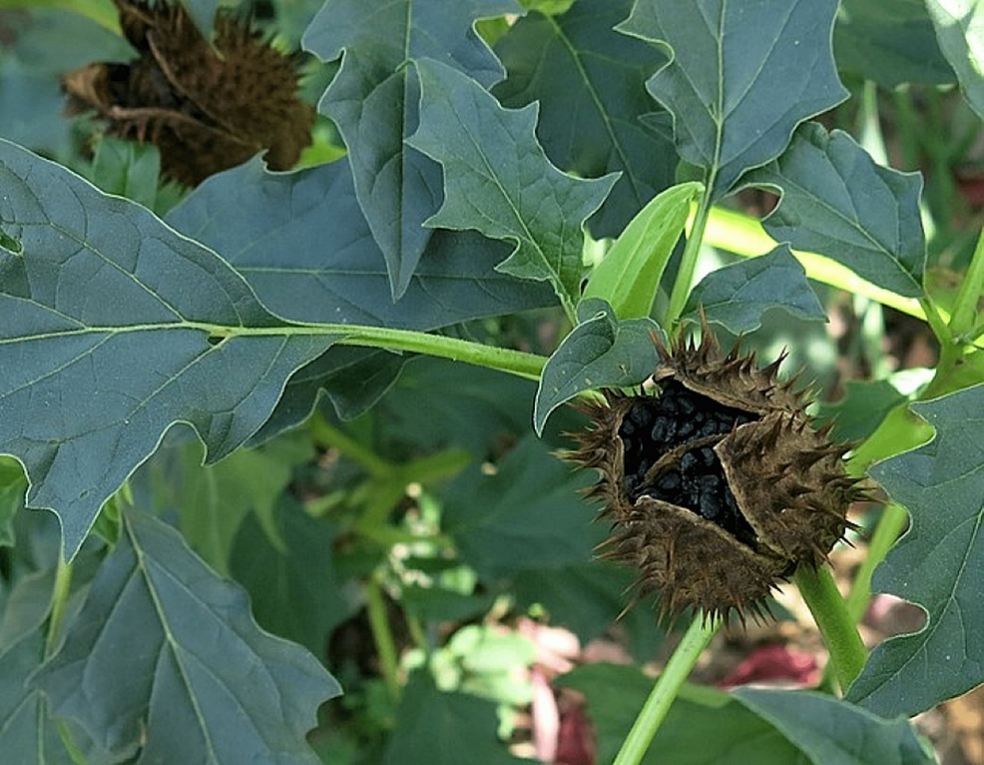
Datura stramonium seed. Adapted from a photo taken by Kostka Martin, September 28, 2015 (https://commons.wikimedia.org/wiki/File:Datura_stramonium_Solanaceae.jpg; public domain).

Distinguished by its large, serrated leaves and trumpet-shaped flowers, Jimson weed can reach a height of up to five feet, adorned with blooms in varying shades of white or purple. Mature plants bear green fruit capsules that house up to a hundred seeds in four compartments. Although the entire plant is toxic, it is the leaves and seeds that contain higher concentrations of atropine, hyoscyamine, and scopolamine. Roughly 6 mg of atropine is found in every 100 seeds, with doses surpassing 10 mg considered potentially lethal (Figure 2) [6].

In contemporary times, Jimson weed poisoning predominantly occurs among adolescents seeking its hallucinogenic effects. In 1998, the American Association of Poison Control Centers documented 152 cases, though the true prevalence is likely higher [7]. The anticholinergic effects of the plant are attributed to its constituents-atropine, hyoscyamine, and scopolamine. Symptoms usually appear within 30-60 minutes post-ingestion and include vivid hallucinations, dry mucous membranes, insatiable thirst, dilated pupils, blurred vision, and difficulties in speech and swallowing. More severe manifestations can involve tachycardia, urinary retention, and ileus. While some cases may progress to hyperthermia, respiratory arrest, and seizures, the slowing of gastrointestinal motility can lead to symptoms lasting 24-48 hours [8].

The characteristic symptoms of anticholinergic toxicity, including mydriasis, dry skin, hallucinations, heightened agitation, urinary retention, and tachycardia, can be summarized as "blind as a bat, dry as a bone, red as a beet, mad as a hatter, and hot as a hare."

Effectively managing Jimson weed poisoning involves critical steps, beginning with initial assessment, symptom recognition, toxin elimination, providing supportive care, and vigilant monitoring. Maintaining open airways, proper breathing, and optimal circulation is vital. In cases of compromised airways, interventions such as intubation and mechanical ventilation may be necessary. A thorough evaluation, along with a detailed medical history, can provide valuable diagnostic clues, especially when Jimson weed poisoning symptoms are not immediately evident. Swiftly recognizing symptoms such as dry mouth, blurred vision, hallucinations, and altered mental status supports early intervention.

To rule out concurrent drug use, toxicology screening is a valuable tool, although it may not always detect pure anticholinergic toxins. Activated charcoal can minimize toxin absorption, and consideration of gastric lavage for removing plant material remains relevant. Inducing emesis is an option for conscious patients. While most cases resolve with vigilant observation, cautious administration of physostigmine, an acetylcholinesterase inhibitor, can counter anticholinergic effects. However, its use carries risks and should be reserved for targeted situations [9]. In addition, benzodiazepines such as diazepam can play a vital role in treatment by addressing acute agitation, and it is crucial to avoid drugs with anticholinergic properties [10].

In conclusion, the harmful effects of Jimson weed exposure can be attributed to its potent anticholinergic agents. Swift recognition, competent supportive care, and sustained monitoring are imperative to ensure patient well-being.

## Conclusions

In summary, Jimson weed poisoning presents with characteristic anticholinergic symptoms, often resolving through supportive care and observation. Swift recognition is key, even though severe cases are infrequent. The profound ingestion of Jimson weed can lead to severe complications, possibly even fatal. Therefore, we should emphasize the necessity of vigilance. Physostigmine stands out as a pivotal treatment for critical instances, while benzodiazepines effectively manage agitation. Furthermore, proactive counseling becomes paramount, particularly during the summer and early fall seasons. This educational effort aims to raise awareness about potential risks, urging individuals to exercise caution when cultivating backyard vegetables. This underscores the importance of informed decision-making regarding consumption, thus fostering a culture of safety and mindfulness. Ultimately, being conscientious about what we grow and consume is an essential step toward a safer and more informed approach to plant-based interactions in our daily lives.

## References

[1] Saunders WB (2007). Emergency management of poisoning. Haddad and Winchester's Clinical Management of Poisoning and Drug Overdose (Fourth Edition).

[2] Centers for Disease Control and Prevention (CDC) (1995). Jimson weed poisoning--Texas, New York, and California, 1994. MMWR Morb Mortal Wkly Rep.

[3] Forrester MB (2006). Jimsonweed (Datura stramonium) exposures in Texas, 1998-2004. J Toxicol Environ Health A.

[4] Vanderhoff BT, Mosser KH (1992). Jimson weed toxicity: management of anticholinergic plant ingestion. Am Fam Physician.

[5] Shenoy RS (1994). Pitfalls in the treatment of jimsonweed intoxication. Am J Psychiatry.

[6] Klein-Schwartz W, Oderda GM (1984). Jimsonweed intoxication in adolescents and young adults. Am J Dis Child.

[7] Tiongson J, Salen P (1998). Mass ingestion of Jimson weed by eleven teenagers. Del Med J.

[8] Rodgers GC Jr, Von Kanel RL (1993). Conservative treatment of jimsonweed ingestion. Vet Hum Toxicol.

[9] Chan K (2002). Jimson weed poisoning—a case report. Perm J.

[10] Spina SP, Taddei A (2007). Teenagers with Jimson weed (Datura stramonium) poisoning. CJEM.

